# Parental Experiences of Administering Pediatric Tuina for Sleep and Appetite in Early School-Aged Children With Attention-Deficit/Hyperactivity Disorder: Qualitative Study in Hong Kong

**DOI:** 10.2196/65471

**Published:** 2025-01-30

**Authors:** Shu-Cheng Chen, Kwai-Ching Lo, Han Li, Pong-Ming Wong, Lok-Yi Pang, Jing Qin, Wing-Fai Yeung

**Affiliations:** 1 Centre for Smart Health, School of Nursing, The Hong Kong Polytechnic University, Hong Kong, China; 2 School of Chinese Medicine, University of Hong Kong, Hong Kong, China; 3 School of Nursing, The Hong Kong Polytechnic University, Hong Kong, China; 4 Research Center for Chinese Medicine Innovation, The Hong Kong Polytechnic University, Hong Kong, China; 5 Research Institute for Smart Ageing, The Hong Kong Polytechnic University, Hong Kong, China

**Keywords:** pediatric massage, child, traditional Chinese medicine, TCM, ADHD, qualitative study, complementary medicine, attention deficit, hyperactivity, massage, *tuina*, *tui na*, mental health, sleep, appetite, parent, parenting, interview, focus group, *anmo*, attention-deficit/hyperactivity disorder

## Abstract

**Background:**

Previous research suggested that parent-administered pediatric *tuina* could improve symptoms of attention-deficit/hyperactivity disorder (ADHD), such as sleep quality and appetite.

**Objective:**

This study aimed to explore the experiences and perceptions of parents administering pediatric *tuina* to school-aged children with ADHD in Hong Kong.

**Methods:**

This qualitative study was embedded in a pilot randomized controlled trial on parent-administered pediatric *tuina* for improving sleep and appetite in school-aged children diagnosed with ADHD. Purposive sampling was used to invite 12 parents who attended a pediatric *tuina* training program and delivered the intervention to their children at home for at least 8 weeks. Data were collected through semistructured focus group interviews and individual interviews, which were audio-recorded, transcribed verbatim, and analyzed using thematic analysis.

**Results:**

Two main themes emerged: (1) effects of parent-administered pediatric *tuina* and (2) parents’ experience of administering pediatric *tuina*. Parents reported significant improvements in children’s sleep quality, appetite, behavior, mental state, and academic performance. Facilitators provided professional guidance and applied a user-friendly course design. Challenges included difficulties in mastering techniques, locating acupuncture points, and time management. Participants suggested the need for more traditional Chinese medicine pattern diagnostic sessions, real-time supervision methods, and extended follow-up to better observe long-term effects.

**Conclusions:**

Parent-administered pediatric *tuina* was perceived to improve children’s sleep quality and appetite significantly, along with other aspects of well-being. Professional guidance and a structured training program facilitated implementation, and challenges highlighted the need for more frequent diagnostic sessions, real-time supervision, and extended follow-up.

## Introduction

### Attention-Deficit/Hyperactivity Disorder

Attention-deficit/hyperactivity disorder (ADHD) is a common neurodevelopmental disorder in children, affecting approximately 5% of the pediatric population [1]. It is characterized by 3 primary symptoms: inattention, hyperactivity, and impulsivity [2,3]. Children with ADHD often experience additional mental, emotional, or behavioral disorders, which can include learning disorders, sleep disorders, oppositional defiant disorders, anxiety, and conduct disorders [4]. Among the comorbidities, sleep problems and appetite disturbances are particularly prevalent among school-aged children with ADHD [5,6]. Sleep problems can include difficulty falling asleep, restless sleep, frequent awakenings, and difficulty waking up in the morning [7]. These sleep issues may be caused by the hyperactive and impulsive nature of ADHD, the side effects of medications, or coexisting emotional and behavioral issues [8]. Eating problems are common in children with ADHD, ranging from poor appetite and picky eating to overeating and cravings for unhealthy foods [9]. The causes of these eating problems can include the side effects of ADHD medications, which often suppress appetite, and the impulsivity associated with ADHD [10]. Notably, stimulant medications, which are commonly prescribed for ADHD, can lead to sleep disturbance and eating problems [11]. Sleeping and eating issues can affect the child’s physical health, growth, and development, which could complicate the management of ADHD symptoms [5,6]. Strategies such as establishing consistent bedtime routines, creating a calming sleep environment, and encouraging balanced diets are crucial to specifically address sleep and appetite issues [5,6]. Parents and caregivers play a vital role in implementing these coping interventions to help manage symptoms and improve the quality of life for the child and the family [12].

### Parent-Administered Pediatric *Tuina*

Pediatric *tuina*, also known as pediatric *anmo* or traditional Chinese medicine (TCM) pediatric massage, is a specialized form of massage therapy tailored for infants and children [13]. It is grounded in TCM principles, which emphasize the harmonious functioning of the body’s systems [13]. In ancient Chinese medicine, the term “double *yang* person” was used to describe individuals exhibiting symptoms of ADHD [14]. By targeting specific acupoints, pediatric *tuina* aims to restore the *yin-yang* balance, thereby enhancing overall health and well-being. As an external, noninvasive therapy, it offers a complementary approach to conventional medical treatments, providing a holistic option for pediatric care [13]. This therapeutic technique has been extensively studied for its potential benefits in addressing various clinical conditions and diseases [15] such as diarrhea [16], anorexia [17], torticollis [18], constipation [19], enuresis [20], and functional dyspepsia [21]. Pediatric *tuina* is used to promote the growth and development of healthy children in China [22]. The practice of pediatric *tuina* involves the stimulation of specific areas or acupoints on the body through various manipulation techniques, including pushing, kneading, pressing, rotating, nipping, circular movements, and pounding [13]. These techniques generate different types of stimuli on the skin, which are detected by surface sensory receptors and transmitted to the central nervous system [23]. This sensory input is believed to induce a series of protective and adaptive homeostatic activities within the body. Research has demonstrated that in young children, the skin can rapidly regulate basic and adaptive homeostatic responses [24]. This regulation may be facilitated by a low compensatory basal level of stress-responsive enzymes, allowing for a broad range of physiological responses [25]. The mechanisms behind these responses suggest that pediatric *tuina* may substantially affect the autonomic nervous system, potentially leading to improved clinical outcomes for various pediatric conditions [26].

### Research Gap

In recent years, several research studies have preliminarily demonstrated the effects of pediatric *tuina* in treating ADHD. For instance, a randomized controlled trial (RCT) on 120 children with ADHD comparing pediatric *tuina* with medication reported that the Conners scores in the pediatric *tuina* group were significantly lower than those in the control group (Cohen *d*=0.96, *P*<.05), as were the ADHD scores (Cohen *d*=.57, *r*=0.28, *P*<.05). The incidence of adverse events was lower in the pediatric *tuina* group (3.33%) than in the control group (16.67%, *P*=.015) [27]. A systematic review of 11 clinical trials suggested the potential benefits of pediatric *tuina* in improving concentration, mood, sleep, and social functioning in children and adolescents with ADHD [28]. However, the extant literature lacks robust qualitative insights into parents’ understanding of using this intervention and the specific implementation as a complementary intervention. Therefore, limited information exists exploring the practicalities, challenges, and perceived benefits of parent-administered pediatric *tuina* for ADHD. The authors’ team recently conducted a pilot RCT to further examine parent-administered pediatric *tuina* for improving sleep and appetite in school-aged children with ADHD in Hong Kong. This study is the qualitative part of the pilot RCT, aiming to further explore parents’ experiences and perceptions of delivering pediatric *tuina* at home, particularly in parents’ experiences on its effects on children’s sleep and appetite and parents’ experience of administering pediatric *tuina*. This project could provide valuable insights into the real-world application of pediatric *tuina* and offer guidance for parents and health care providers in optimizing this intervention’s use in more contexts.

## Methods

### Ethical Considerations

Ethical approval for the study was granted by the Hong Kong Polytechnic University (HSEARS20230810005). A written informed consent to participate in this study was obtained from the participants. Each participant was assigned a randomly generated code to ensure confidentiality. Each participant received a cash incentive of HK $200 (approximately US $25.64 based on an exchange rate of HK $1=US $0.1282) to acknowledge their participation.

### Study Design

**Figure 1.**
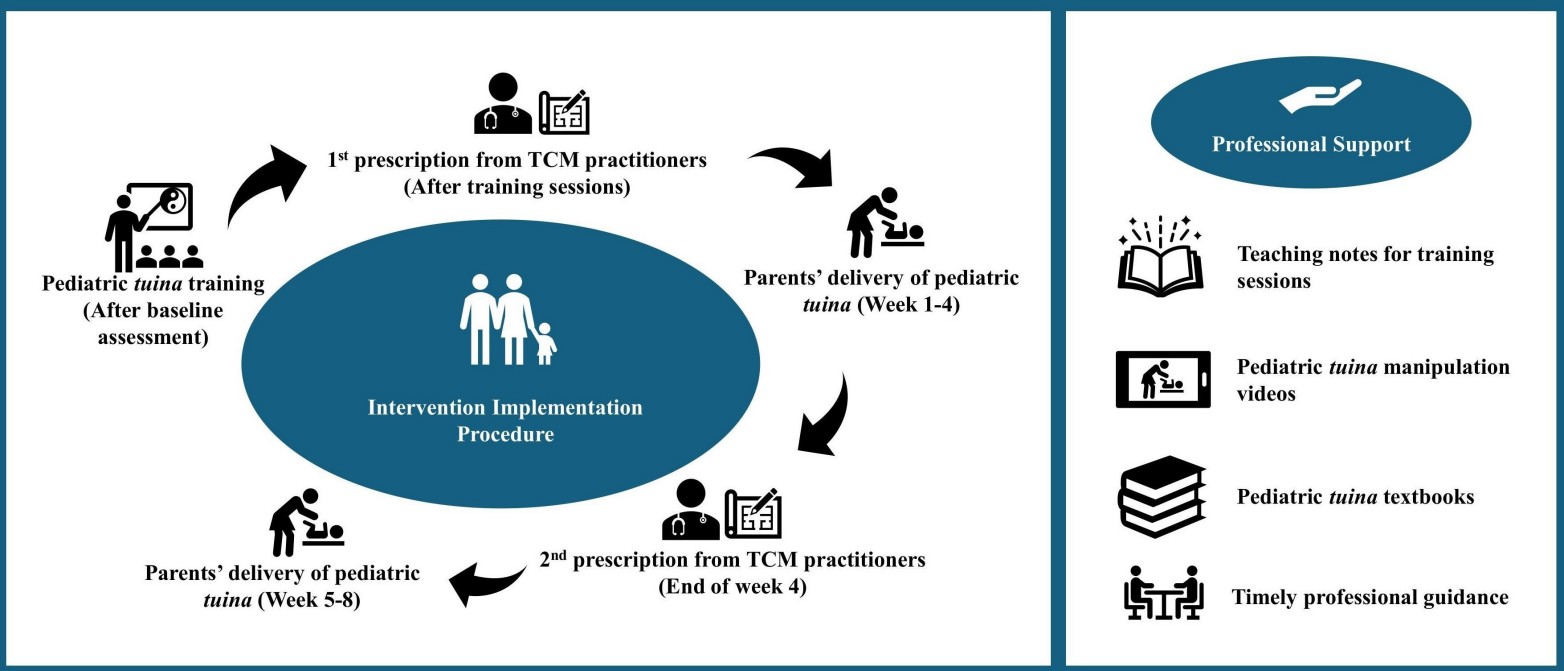
The implementation of the parent-administered pediatric *tuina* program. TCM: traditional Chinese medicine.

This project was registered on ClinicalTrials.gov under the identifier NCT06007742. This study presents the qualitative findings from a pilot RCT investigating the effects of parent-administered pediatric *tuina* on sleep quality and appetite in school-aged children with ADHD in Hong Kong. In the RCT, parents received systematic training from TCM practitioners, including 2 face-to-face sessions (each lasting 2 hours). The first session focused on theoretical knowledge, and the second session covered TCM pattern identification and pediatric *tuina* techniques. Following the training sessions, parents administered pediatric *tuina* to their children at home over an 8-week period on the basis of individualized prescriptions formulated by TCM practitioners. Each participant in the pediatric *tuina* group received parent-administered pediatric *tuina* sessions every other day, totaling 24 sessions over an 8-week period (at least 3 sessions each week). Each session lasted approximately 25‐30 minutes. The *tuina* protocol was developed from a 2021 feasibility RCT for ADHD involving 64 parent-child pairs, where 128 individualized pediatric *tuina* prescriptions were analyzed to identify commonly used and safe acupoints [29]. The resulting standardized basic prescription allows TCM practitioners to make individualized modifications based on syndrome differentiation. This TCM diagnosis guided the selection of specific acupoints and manipulation techniques tailored to each child’s condition. Details regarding the implementation of the intervention are illustrated in Figure 1. The interviews commenced 1 week after the completion of treatment for the first wave of participants in the RCT, which was conducted in 3 sequential waves. Interviews were strategically scheduled at 3 distinct timepoints, following the conclusion of each treatment wave. This timing ensured that participants had sufficient experiences and perceptions for in-depth exploration and allowed for the prompt collection of data while their experiences were still vivid, ensuring accurate and immediate reflections on the treatment effects. The study’s reporting adhered to the Consolidated Criteria for Reporting Qualitative Research’s checklists [30].

### Setting

This study used a qualitative approach with the use of semistructured focus group interviews and individual interviews. The research methodology allows for a great flexibility of the participants to join. The focus group interviews were conducted face-to-face in the campus of the Hong Kong Polytechnic University.

### Participants

Participants were parents with children with ADHD. The participants for the interviews were invited from the participants in the pilot RCT who received the parent-administered pediatric *tuina* intervention [31]. Recruitment information was sent to potential participants by WhatsApp message at the end of February 2024, and purposive sampling was applied for recruitment. The children included in the pilot RCT (1) were aged 6‐8 years; (2) possessed internationally recognized diagnostic information or certification for ADHD; and (3) had a score equal to or higher than 39 (borderline cutoff) of the Sleep Disturbance Scale for Children, which indicated sleep problems of children. The parents included in this study (1) participated in the project on using parent-administered pediatric *tuina* for improving sleep quality and appetite in children with ADHD who completed the 2-month treatment and follow-up assessment of the intervention during the treatment period, (2) were able to communicate in Cantonese fluently, and (3) agreed to participate and were willing to share their experience in applying this intervention. This study focused on children aged 6‐8 years, representing the early school-age period. This study focused on children aged 6‐8 years, representing the early school-age period. This focus is driven by the significance of early intervention in ADHD’s developmental trajectory and the specific responsiveness of this age group to pediatric *tuina*. Research suggests that early school years are critical for implementing interventions that could substantially alter the course of ADHD, making timely and targeted intervention essential [32]. Furthermore, tactile therapies such as pediatric *tuina* are shown to be highly effective in younger children, who are generally more receptive to such treatments [33].

### Data Collection

A semistructured interview guide was created and refined on the basis of previous relevant studies and comments from experts, including 2 TCM practitioners (KCL and PMW) and 2 qualitative researchers (WFY and SCC). The guide comprised 5 open-ended questions detailed in Textbox 1. The focus group interviews were moderated by the first author (SCC), with an assistant moderator (LYP) responsible for note-taking and operating the recording equipment. Both moderators received training from the corresponding author (WFY), a TCM practitioner with extensive research experience in TCM interventions and qualitative methodologies. At the beginning of each interview session, the moderator introduced herself, clarified the study’s purpose and procedures, and emphasized the confidentiality rules. She also explained the questions and facilitated group interaction by providing prompts and pauses, ensuring that the discussion remained focused without imparting any value judgments. Participation in the study was voluntary, and no individuals other than the participants and researchers were present during the interviews. Participants were reassured that, although the sessions were audiotaped, their names would not be recorded.

Textbox 1. **Questions for the semistructured interview.**

Do you think pediatric *tuina* has changed or affected your child’s eating habits?

Do you think pediatric *tuina* has changed or affected your child’s sleep?

Besides diet and sleep, in what other aspects do you think pediatric *tuina* has changed or affected your child?

What difficulties did you encounter from learning to performing pediatric *tuina*?

How do you evaluate the content and process of the pediatric *tuina* treatment for attention-deficit/hyperactivity disorder? What are the advantages and areas for improvement?

### Data Analysis

All interviews were audiotaped and transcribed verbatim in traditional Chinese before data analysis commenced. All study-related documents and transcripts were deidentified, and the audio files were destroyed once transcription was completed. The transcripts were analyzed using thematic analysis with hierarchical coding [34,35]. The template analysis applied the following steps: (1) an initial reading of the transcripts to identify a priori themes and perform preliminary coding; (2) development of an initial coding template; (3) systematic review of all datasets to refine the template by adding, removing, or merging codes as necessary; and (4) finalization of the template for application to the entire dataset [36]. The first author (SCC) conducted the transcription and initial coding, which were subsequently reviewed for accuracy and consistency by another researcher (HL). Any discrepancies in coding were resolved through consultation with the principal investigator (WFY). Microsoft Word software was used to manage the coding process [37,38]. Descriptive statistics were used to summarize the demographic characteristics of the sample. The Results section presented a summary of the main themes and subthemes, illustrated with participant quotes. For each quote, only the participant codes are provided.

### Trustworthiness

This study adhered to the trustworthiness criteria for qualitative research as outlined by Lincoln and Guba [39,40]. For credibility, a semistructured interview guide was developed through 2 group discussions and subsequently pilot-tested during the initial interview session. Purposive sampling was used to select participants capable of providing diverse perspectives and experiences relevant to the intervention delivery. For transferability, the richness of the data was assessed using the saturation theory [41]. Data collection continued until the point of near exhaustion of new information, which was achieved by the third session [42]. Dependability was maintained through independent coding of the collected data by 2 researchers, complemented by regular debriefing sessions with WFY, an individual with substantial expertise in qualitative research. Finally, participants were provided with the findings, including the code tree and quotations, for their feedback and verification to ensure confirmability. This process aimed to minimize bias and ensure that the intervention reflected the true perspectives and experiences of the participants.

## Results

### General Characteristics of Data

In total, 4 group interviews and 3 individual interviews were conducted in Cantonese between February 2024 and April 2024. The group interviews averaged 86 minutes in duration, ranging from 77 to 96 minutes, and the individual interviews averaged 35.3 minutes, with durations ranging from 30 to 46 minutes. Of the 61 parents invited, 12 ultimately participated. Each interview session included between 1 and 3 participants, none of whom knew each other. Themes and subthemes were initially identified by the fourth interview session and further refined by the seventh session.

### Sample Profile

Overall, 12 parents, comprising 10 females (83.3%) and 2 males (16.7%), whose children were diagnosed with ADHD participated in the interviews. The average age of the parents was 40.1 (SD 3.7) years, and the mean age of their children was 6.9 (SD 0.9) years. Detailed demographic information on the participants can be found in Table 1.

**Table 1.** Demographic characteristics of participants interviewed (N=12).

Characteristics		Values
**Age (years), mean (SD)**		
	Parents	40.1 (3.7)
	Child	6.9 (0.9)
**Gender (parent), n (%)**		
	Male	2 (16.7)
	Female	10 (83.3)
**Gender (child), n (%)**		
	Male	10 (83.3)
	Female	2 (16.7)
**Educational level (parent), n (%)**		
	Senior high school	4 (33.3)
	College or above	8 (66.7)
**Career (parent), n (%)**		
	Professional/semiprofessional	4 (33.3)
	Unskilled worker	1 (8.3)
	Homemaker	5 (41.7)
	Others	2 (16.7)
Family members, mean (SD)		4.3 (1.1)
**Family monthly income in HK$^a^, n (%)**		
	10,001‐24,999	2 (16.7)
	25,000‐49,999	7 (58.3)
	50,000 or above	3 (25)
BMI (child), mean (SD)		14.3 (1.8)
**Past treatment for ADHD**^b^ **(child), n (%)**		
	Medication	0 (0)
	Cognitive behavioral therapy	3 (25)
	Others	1 (8.3)
**Current treatment for ADHD (child), n (%)**		
	Medication	3 (25)
	Cognitive behavioral therapy	3 (25)
	Others	1 (8.3)

a All income values are presented in Hong Kong dollars. For the purpose of this study, the conversion rate used is HK $1=US $0.1282 (as of February 2024).

b ADHD: attention-deficit/hyperactivity disorder.

### Major Themes

Two themes regarding the participants’ experience in applying the parent-administered pediatric *tuina* intervention and participating in the study were identified: (1) effects of parents performing pediatric *tuina* and (2) parents’ experience of performing pediatric *tuina*. The specific subthemes under each theme were described. Table 2 presents the code structure.

**Table 2.** Code structures.

Themes and subthemes		Code units
**Effects of parent-administered pediatric** ***tuina***		
	Improvements in children’s eating	Increased appetite and food intake Improved diet structure (less picky eating and trying new things) Improved gastrointestinal function (indigestion, vomiting, and constipation)
	Improvements in children’s sleep	Improved sleep quality (restlessness, snoring, teeth grinding, sweating, and yelling) Improved sleep habits (shorter time to fall asleep and able to sleep on their own)
	Improvements in other aspects of children	Improved behavioral habits (milder behavior) Improved mental state (relaxed and better emotions) Improved attention (academic performance) Improved interpersonal relationships (family and school) Improved physical condition (bedwetting, rhinitis, weight, and height)
**Parents’ experience of performing pediatric** ***tuina***		
	Advantages of this intervention implementation	User-friendly course design (appropriate difficulty and clear teaching materials) Professional guidance from instructors Noninvasive treatment method Customized diagnosis and treatment Satisfactory treatment effects (effective and quick results)
	Difficulties encountered during implementation	Difficulty in operation (knowledge, locating acupuncture points, and techniques) Difficulty in children’s cooperation Time management difficulties (parents and children)
	Parents’ suggestions on improving the intervention	Increasing guidance during practice and operation Use of real-time supervision methods (such as electronic records or apps) Increasing number of diagnostic sessions Extending treatment and follow-up time

### Effects of Parent-Administered Pediatric *Tuina*

This theme encompasses three subthemes: (1) improvements in children’s eating, (2) improvements in children’s sleep, and (3) improvements in other aspects of children.

#### Improvements in Children’s Eating

Parents observed noticeable improvements in their children’s eating habits following the administration of pediatric *tuina*. Many reported an increase in appetite and food intake, as illustrated by one parent who stated, “I think his appetite is better...at least he’s willing to eat more, which is an achievement; the most important thing is that he’s willing to eat” [Participant 12]. Another parent noted a reduction in the child’s resistance to eating: “Normally, if we hurry him to eat, he immediately says he’s full, but after doing *tuina*, he says he wants more, and his food intake has increased” [Participant 59]. Additionally, some parents observed an improvement in their children’s diet structure, with children becoming less picky and more willing to try new foods. A parent shared, “After the pediatric *tuina*, I felt he started eating meat...he even ate fish that he previously didn’t eat” [Participant 1]. Improvements in gastrointestinal function were reported, with one parent noting a remarkable change: “His stools have clearly improved, previously they were like small pellets, similar to Maltesers...during the massage period, his stools became much more normal” [Participant 50]. Another parent highlighted the benefits of stomach massages: “I think massaging his stomach helped with bowel movements...his stomach is more relaxed now, it used to be tight, and his belly was cold before, now it’s warm” [Participant 40].

#### Improvements in Children’s Sleep

Parents also reported enhancements in their children’s sleep quality and habits after pediatric *tuina*. Improved sleep quality was a common theme, with one parent describing their child’s more stable sleep: “Before doing *tuina*, he used to sleep like a whirlwind, rolling from the head to the foot of the bed and back all night long, but now he’s more stable and it’s less frequent” [Participant 59]. Another parent mentioned a reduction in excessive sweating and sleep disturbances: “He usually sweats a lot while sleeping, even at 4 AM he still sweats, but after the *tuina*, he sweats less...also, he used to yell in his sleep, I had to wake him, about two or three times a week, but this has noticeably reduced now” [Participant 50]. Improved sleep habits, such as shortened time to fall asleep and the ability to sleep independently, were noted. A participant shared, “He sleeps more soundly now; previously, he had no sense of security and needed me to accompany him to sleep, but now, for example, after I massage him and turn off the lights, he is willing to sleep by himself, at least it’s the first step” [Participant 40]. Another mom observed, “It takes him less time to fall asleep now; it used to take him two hours to fall asleep, but now it might only take half an hour” [Participant 59].

#### Improvements in Other Aspects of Children

Beyond eating and sleep, parents observed various other benefits of pediatric *tuina* on their children’s behavior and overall well-being. Improvements in behavioral habits were frequently mentioned, with one parent noting a decrease in aggressive behavior: “I think he has fewer outbursts, and so do I...he used to hit people, very bad temper like a volcano...now I feel like he has fewer explosive moments” [Participant 12]. Enhancements in the children’s mental state were reported, with one mom describing their child as more relaxed: “I think he’s more relaxed, when I massage him, I ask if he likes it, if it hurts...he says he likes it, it’s very comfortable...I think his emotions are more relaxed” [Participant 43]. Another parent noticed improved emotional expression: “Previously, he would get very angry if he didn’t like what I said, but now he just says, ‘Mom, I don’t like it, I don’t want you to say that,’ expressing himself more gently” [Participant 13]. Improvements in attention and academic performance were highlighted by some parents, with one noting, “The homeroom teacher says he’s doing okay, although he still gets easily distracted sometimes, but compared to last semester, he has made progress” [Participant 40]. Another parent observed remarkable academic improvements: “My son scored over 70 in listening in the first semester, but after doing *tuina*, he scored 95 in both Chinese and English exams at the end of March; I’m not sure if it had an effect, but I can see his attention has improved” [Participant 52]. Enhanced interpersonal relationships were reported, with one parent stating, “During the *tuina* process, the parent-child relationship improved, he likes to discuss math with his dad, using many methods to calculate, which helps his academics, he feels a sense of achievement, more confident, and even teaches friends how to calculate, and his relationship with classmates has improved” [Participant 15]. Physical conditions, such as bedwetting, rhinitis, weight, and height, were noted to have improved. One parent shared, “After the *tuina*, his nose is no longer as sensitive, it used to be really bad, I even thought about taking him to see a doctor” [Participant 52]. Another parent observed, “Her physical condition has really improved, he eats more, sleeps better, and her weight has increased...she gained a few pounds compared to last semester” [Participant 52]. Bedwetting was reported to have decreased, with one parent stating, “He used to wet the bed, but recently it has decreased” [Participant 43].

### Parents’ Experience of Performing Pediatric *Tuina*

Parents’ experiences of administering pediatric *tuina* include three subthemes: (1) advantages of this intervention implementation, (2) difficulties encountered during implementation, and (3) parents’ suggestions on improving the intervention.

#### Advantages of This Intervention Implementation

Parents highlighted several advantages of implementing pediatric *tuina* for their children. One notable advantage was the user-friendly course design, which many found to be appropriately challenging yet accessible. As one parent explained, “The Chinese medicine doctor starts by explaining things, we initially didn’t know much about acupuncture points, but I think the course depth is suitable for us parents” [Participant 31]. Additionally, parents appreciated the professional guidance from instructors, with another parent noting, “I think they did a good job. A Chinese medicine PhD analyzed the problem, and then a *tuina* therapist taught the techniques and let me record videos. After filming, they explained the whole process, answered my questions directly, and didn’t make it difficult to grasp. Later, they sent me the technique videos, which was great” [Participant 13]. The noninvasive nature of pediatric *tuina* was valued, with one parent stating that “(Pediatric *tuina*) is more natural and noninvasive, which is already very good” [Participant 1]. Furthermore, parents appreciated the customized diagnosis and treatment plans tailored to their children’s specific needs. One parent shared, “The first time I came back to see the doctor, I described my child’s situation, and the doctor added two acupuncture points, explaining that it was because of my child’s current condition, which I found acceptable” [Participant 43]. Lastly, the satisfactory treatment effects were highlighted, with parents observing quick improvements in their children’s overall well-being. As one parent noted, “I think pediatric *tuina* is effective for my child. At least his emotions improved, as well as his appetite, diet, growth, and digestion” [Participant 1]. Another parent remarked, “After two weeks, I noticed he slept better” [Participant 59], and another one observed, “In the first week, I felt he became more obedient and focused” [Participant 4].

#### Difficulties Encountered During Implementation

Despite the advantages, parents also encountered several difficulties during the implementation of pediatric *tuina*. One common challenge was the difficulty in operation, particularly in terms of knowledge, locating acupuncture points, and mastering techniques. As one parent expressed, “When actually performing the *tuina*, we were guessing. I felt uncertain about the position and pressure, and we were trying to imitate, so some areas were unclear, like using several fingers...” [Participant 43]. Another difficulty was gaining children’s cooperation, with one parent noting, “He found repeating the same thing boring and asked if there was anything else to do” [Participant 40]. Time management posed a considerable challenge for parents and children. One parent mentioned: “I was very busy myself and didn’t have the determination to schedule a specific time for the *tuina*” [Participant 13]. Another parent added, “If I felt it was too late or he was tired that day, I would do one or two techniques and then sleep” [Participant 40].

#### Parents’ Suggestions on Improving the Intervention

Parents suggested several improvements to enhance the implementation of parent-administered pediatric *tuina*. Increased guidance during practice and operation was a common request. One parent suggested, “It would be better if the doctor could watch the whole process of how I execute it from start to finish. We are beginners and may not notice if we are making mistakes” [Participant 43]. Another parent proposed: “I hope the doctor can check our techniques after a few weeks” [Participant 24]. The use of real-time supervision methods, such as electronic records or applications, was suggested to facilitate the process. As one parent noted, “The paper (logbook) could be converted to phone input, as *tuina* requires using olive oil, and the paper gets oily after filling it out” [Participant 50]. Another parent mentioned, “Using an app would save parents some effort. Besides recording if the points were done, it could also record the time, duration, and timely track the child’s weight, eating, bowel movements, and sleep” [Participant 31]. Parents also expressed a desire for an increased number of diagnostic sessions. One parent stated, “More TCM pattern diagnostic sessions would give us more confidence. Now it’s once every four weeks; if it were every two weeks, we’d feel more assured. Sometimes we are just blindly following instructions” [Participant 59]. Lastly, extended treatment and follow-up time were deemed necessary by some parents to observe long-term effects. As one parent remarked, “I think it needs more follow-up, a few months would be best. I genuinely want to see the long-term effects” [Participant 52].

## Discussion

### Main Findings

This study explored the effects and parental experiences of administering pediatric *tuina* to improve sleep and appetite in school-aged children with ADHD. This study is the first qualitative investigation into parent-administered pediatric *tuina* for addressing specific issues in children in Hong Kong. Insights from 12 parents were gathered using semistructured focus group interviews and individual interviews. The findings revealed 2 key themes: the effects of pediatric *tuina* on children and parents’ experiences with the intervention. Parents reported significant improvements in their children’s eating habits, sleep quality, and other areas such as behavior, mental state, and academic performance. They also highlighted advantages such as the user-friendly course design and professional guidance but noted challenges in mastering techniques and managing time. Parents suggested more guidance, real-time supervision, frequent diagnostic sessions, and extended follow-up to improve the intervention.

### Comparison With Previous Studies

The findings of this study align well with a previous RCT on the effects of pediatric *tuina* for improving children’s sleep quality and habits, eating habits, behavioral regulation, emotional well-being, and parent-child relationship [29]. Improvements on children’s sleep quality and habits are consistent with the results of several previous studies examining the effects of pediatric *tuina* on sleep disturbance [43,44] or sleep problems in populations with different conditions such as bronchitis [45] and pneumonia [46]. The enhancements in eating habits reported by parents are in line with findings from a systematic review of pediatric *tuina* for anorexia in children on 28 RCTs [17]. In this study, a meta-analysis based on 9 RCTs indicated that pediatric *tuina* was superior to Western medicine (mean difference: −0.88, 95% CI −1.27 to −0.5) and Chinese herbs (mean difference: −0.69, 95% CI −1 to −0.38) in terms of improving food intake, suggesting that pediatric *tuina* could be an effective intervention for children with eating difficulties. The improvements in behavioral regulation and emotional well-being noted in this study mirror those documented in research on other pediatric massage therapies. Previous studies have found that children receiving massage therapy exhibit lessened behavioral outbursts, improved self-regulation, enhanced emotional regulation, reduced anxiety, and improved mood [47,48]. Moreover, the findings regarding the enhancement of parent-child relationships resonate with previous research emphasizing the importance of parental involvement in therapeutic outcomes. Studies on parent-involved therapies, such as cognitive behavioral therapy for children with ADHD, have shown that active parental participation is crucial for achieving effective outcomes [49,50]. Parent-administered pediatric *tuina* increases quality time spent between parents and children, thus fostering closer bonds, enhanced communication, and a sense of security. This engagement is similar to the benefits seen in cognitive behavioral therapy, where parental involvement plays a critical role in the success of the intervention.

The findings reveal notable inconsistencies with previous research focused on the effects of parent-administered pediatric *tuina* on children’s attention. While a prior qualitative study on exploring the effects of parent-administered pediatric *tuina* on ADHD in preschool children conducted in Mainland China reported that almost all parents who participated reflected that pediatric *tuina* had minimal effect on improving attention in their children [51], this study reveals considerable improvements in children’s attention and academic performance based on the description from several parents. The possible explanation may be attributed to the context (eg, culture and intervention implementation) and population differences between the two studies. In this study, the participants are school-aged children who may be more responsive to physical touch due to their developmental stage [52] and the academic demands [53] they face, which could enhance the effect of improved attention on academic performance. Additionally, cultural factors in Hong Kong, such as parental involvement and parental warmth in education, may have contributed to the more pronounced effects observed in this study [54]. Besides, the face-to-face TCM pattern diagnosis and parent training of this study may be more accurate than the web-based intervention implementation mode of the previous study, thereby generating more satisfactory effects on children’s attention. These inconsistencies underscore the need for further research to explore the contextual factors that influence the implementation of pediatric *tuina* across different populations and developmental stages.

One unanticipated finding was that pediatric *tuina* produced remarkably fast-acting benefits across various health outcomes, as reported by parents. For instance, one parent observed that their child’s sleep quality improved noticeably after just 2 weeks of treatment. Another parent noted that their child’s weight, which had been stagnant at 16 or 17 kg for a year, increased rapidly to 18 kg after 4 weeks of pediatric *tuina* therapy. Additionally, another parent observed considerable behavioral changes within the first week, noting that their child became more obedient and focused. Possible explanations for the fast-onset of pediatric *tuina* relate to the young age of participants and the underlying effect theory of pediatric *tuina*. According to the TCM theory, children, particularly those under 6 years of age, are more responsive to sensory stimuli due to their more sensitive organs [23], making them more receptive to external manipulations. This heightened sensitivity potentially allows the effects of pediatric *tuina* to activate the body’s self-healing mechanisms quickly [55]. The concentration of pediatric *tuina* acupoints in areas rich in sensory receptors, such as the palms and head, enables effective stimulation of these receptors, thereby initiating quick physiological responses [56]. Furthermore, the specific manipulations used in pediatric *tuina*, such as pressing and rubbing, provide varied sensory stimuli that are rapidly processed by the nervous system, leading to swift improvements in the body [26]. While previous research has primarily focused on the targeted outcomes of pediatric *tuina*, the findings of this study offer new insights into the onset speed of these benefits, challenging the common perception that TCM interventions are primarily suited for chronic conditions and work slowly.

### Implications

The findings have remarkable implications for several key stakeholders. For pediatricians, the integration of pediatric *tuina* into conventional ADHD treatment protocols is recommended, particularly for managing common comorbidities such as sleep and eating disorders in young children. For TCM practitioners, developing systematic training programs for parents to administer pediatric *tuina* effectively at home is meaningful for extending the benefits of this therapy beyond clinical settings and addressing a broader range of pediatric conditions. For researchers focusing on parent-administered pediatric *tuina*, future clinical trials should aim to enhance the guidance provided during practice, which can be achieved by developing real-time supervision methods, increasing the frequency of diagnostic sessions, and extending the treatment and follow-up periods. Besides, future research should consider triangulating qualitative findings with quantitative data to enhance the robustness and applicability of the study results.

### Limitations

The limitations of this qualitative study should be acknowledged. First, the mixed-method approach, incorporating individual interviews and focus group interviews, was necessitated by time management barriers faced by participants. While this approach allowed great flexibility and participation, it may have introduced variations in the depth and breadth of data collected, potentially affecting the consistency and comparability of the findings. Second, the study was geographically limited to Hong Kong, which may have restricted the generalizability of the results to other populations and cultural contexts. The unique cultural and health care practices in Hong Kong could have influenced parents’ perceptions and experiences, potentially differing considerably from those in other regions. Third, only participants who were willing to attend and had completed the intervention period were included, which may have excluded the perspectives of those who declined to participate in the interviews and may have expressed more negative views.

### Conclusion

The findings reveal that parent-administered pediatric *tuina* considerably improved children’s sleep quality, appetite, behavioral habits, mental state, and academic performance. Parents appreciated the professional guidance and user-friendly course design, which facilitated the intervention’s implementation. However, challenges included difficulties in mastering techniques, locating acupuncture points, and managing time. Parents expressed the need for more frequent diagnostic sessions, real-time supervision, and extended follow-up to observe the long-term effects. Future research should address these challenges and consider integrating qualitative findings with quantitative data to enhance the robustness and applicability of the results.

## Abbreviations

ADHD: attention-deficit/hyperactivity disorder
RCT: randomized controlled trial
TCM: traditional Chinese medicine
